# *MCP1* SNPs and Pulmonary Tuberculosis in Cohorts from West Africa, the USA and Argentina: Lack of Association or Epistasis with *IL12B* Polymorphisms

**DOI:** 10.1371/journal.pone.0032275

**Published:** 2012-02-27

**Authors:** Digna R. Velez Edwards, Alessandra Tacconelli, Christian Wejse, Philip C. Hill, Gerard A. J. Morris, Todd L. Edwards, John R. Gilbert, Jamie L. Myers, Yo Son Park, Martin E. Stryjewski, Eduardo Abbate, Rosa Estevan, Paulo Rabna, Giuseppe Novelli, Carol D. Hamilton, Richard Adegbola, Lars Østergaard, Scott M. Williams, William K. Scott, Giorgio Sirugo

**Affiliations:** 1 Dr. John T. Macdonald Foundation Department of Human Genetics and Miami Institute of Human Genomics, University of Miami, Miami, Florida, United States of America; 2 Center for Human Genetics Research, Vanderbilt University, Nashville, Tennessee, United States of America; 3 Vanderbilt Epidemiology Center, Institute of Medicine and Public Health, and Department of Obstetrics and Gynecology, Vanderbilt University Medical Center, Nashville, Tennessee, United States of America; 4 Centro di Genetica, Centro di Ricerca Scientifica, Ospedale San Pietro FBF, Rome, Italy; 5 Bandim Health Project, Danish Epidemiology Science Centre and Statens Serum Institute, Bissau, Guinea-Bissau; 6 Department of Infectious Diseases, Aarhus University Hospital, Skejby, Denmark; 7 Center for Global Health, School of Public Health, Aarhus University, Skejby, Denmark; 8 MRC Laboratories, Fajara, The Gambia (West Africa); 9 Centre for International Health, University of Otago School of Medicine, Dunedin, New Zealand; 10 Division of Epidemiology, Department of Medicine, Vanderbilt Epidemiology Center, Vanderbilt University School of Medicine, Nashville, Tennessee, United States of America; 11 Division of Infectious Diseases, Department of Medicine, Centro de Educación Médica e Investigaciones Clínicas “Norberto Quirno” (CEMIC), Buenos Aires, Argentina; 12 Department of Medicine, Hospital F. J. Muñiz, Buenos Aires, Argentina; 13 Dipartimento di Biopatologia e Diagnostica per Immagini, Università di Tor Vergata, Rome, Italy; 14 Family Health International 360, Research Triangle Park, North Carolina, United States of America; 15 Duke University Medical Center, Durham, North Carolina, United States of America; 16 Bill & Melinda Gates Foundation, Seattle, Washington, United States of America; Fundació Institut d′Investigació en Ciències de la Salut Germans Trias i Pujol - Universitat Autònoma de Barcelona - CIBERES, Spain

## Abstract

The monocyte chemotactic protein-1 (MCP-1) is a chemokine that plays an important role in the recruitment of monocytes to *M. tuberculosis* infection sites, and previous studies have reported that genetic variants in *MCP1* are associated with differential susceptibility to pulmonary tuberculosis (PTB). We examined eight *MCP1* single nucleotide polymorphisms (SNPs) in a multi-ethnic, case-control design that included: 321 cases and 346 controls from Guinea-Bissau, 258 cases and 271 controls from The Gambia, 295 cases and 179 controls from the U.S. (African-Americans), and an additional set of 237 cases and 144 controls of European ancestry from the U.S. and Argentina. Two locus interactions were also examined for polymorphisms in *MCP1* and interleukin 12B (*IL12B*), another gene implicated in PTB risk. Examination of previously associated *MCP1* SNPs rs1024611 (−2581A/G), rs2857656 (−362G/C) and rs4586 (+900C/T) did not show evidence for association. One interaction between rs2857656 and *IL12B* SNP rs2288831 was observed among Africans but the effect was in the opposite direction in Guineans (OR = 1.90, p = 0.001) and Gambians (OR = 0.64, p = 0.024). Our data indicate that the effect of genetic variation within *MCP1* is not clear cut and additional studies will be needed to elucidate its role in TB susceptibility.

## Introduction

Approximately one third of the world's population is infected with *Mycobacterium tuberculosis* (*Mtb*) with a global burden of TB disease in 2009 of 9.4 million incident cases, 14 million prevalent cases and more than 1.6 million deaths. According to the 2010 WHO global report on TB, the great majority of cases were in the South-East Asia, Africa and the Western Pacific (35%, 30% and 20%, respectively). An estimated 11–13% of incident cases were HIV-positive and approximately 80% of these cases were in Africa [1]. However, the majorities of those infected with *Mtb* maintain a latent state and do not convert to clinical disease but do remain at risk of progressing to active TB later. Factors that can modulate progression to active TB include gender, anemia, smoking and alcohol consumption as well as bacterial and host genetic factors [2]–[4]. In addition, increasing rates of TB and HIV have been highly correlated and a large percentage of TB cases are HIV-positive. Nonetheless, a substantial proportion of risk remains unexplained [5].

Evidence from human and animal studies indicates that *Mtb* clearance is genetically regulated [6]. Twin studies, genome-wide linkage and association analyses as well as candidate gene studies support the notion that human genetic factors play a role in the development of TB [7]–[9] and the majority of genes that have been implicated so far are in immunological pathways [8]. However, studies implicating genetic loci or specific genes have sometimes been inconsistent, possibly due to heterogeneity in phenotype definitions and study populations. Such heterogeneities are common challenges in global studies of etiologically complex traits, such as risk for *Mtb* infection and progression to TB [10].

The chemokine (C-C motif) ligand 2/monocyte chemotactic protein 1 gene (*CCL2/MCP1*) encodes the CCL2/MCP-1 protein, a member of the CC chemokine subfamily that is characterized by a two cysteine residue motif proximal to the amino-terminus of the protein. The MCP-1 chemokine plays a key role in the granulomatous reaction in lung tissue and *Mtb* containment in mouse models occurs through an interaction with the cognate receptor, chemokine (C-C motif) receptor 2 (CCR2), expressed on monocytes, macrophages, CD4+ T cells and immature dendritic cells [11]. The chemokine MCP-1 was associated with severe tuberculosis and was proposed as a marker of disease severity [12].

The 17q11-q21 chromosomal region encompassing *MCP1* was initially identified as a candidate for TB susceptibility in linkage analyses of multi-case tuberculosis and leprosy families from Brazil and the critical interval was subsequently refined to 17q11.12 [13]. An *MCP1* promoter variant has been associated with increased susceptibility to pulmonary TB (PTB), which is mediated through the inhibition of cytokine IL-12p40 production and is required for IFNγ-induced protection from PTB. Functional studies by Flores-Villanueva et al. showed that the GG genotype at *MCP1* “−2518” (alias −2581 used in this paper, rs1024611) had the highest MCP-1 plasma levels and lowest IL-12p40 plasma concentrations in TB patients [14]; IL-12p40 (encoded by *IL12B*) is required for IFNγ-induced protection from PTB and the GG homozygotes had 5× higher odds of developing TB than AA homozygotes [14]. The same SNP has also been associated with modulation of risk for spina bifida, coronary artery disease, and HIV-1, suggesting that rs1024611 could be a pleiotropic mutation with effects on different but key biological pathways [15]–[17].

Based on the potential biological role of *MCP1* in PTB susceptibility and previous evidence implicating this gene, we undertook this study to investigate the association of gene variants of *MCP1* with susceptibility to PTB in two West African populations (Guinea-Bissau and The Gambia) and to replicate the results in African-Americans and samples of European ancestry from North and South America. We assayed eight SNPs in *MCP1* in DNA samples from 321 PTB cases and 346 controls from Guinea-Bissau, 258 PTB cases and 271 controls from The Gambia, 295 cases and 179 controls that are African-Americans, and 237 cases and 144 controls of European ancestry from North and South America. Genetic data were evaluated for association with PTB risk. In addition, motivated by our previous findings of an association between *IL12B* and PTB and the known biological interaction between the gene products, we assessed whether polymorphisms in *IL12B* modify PTB susceptibility due to *MCP1* variation [18].

## Materials and Methods

### Study Populations

Detailed clinical and demographic information for subjects from Guinea-Bissau, The Gambia, the United States (African-Americans) and North and South Americans of European ancestry has been previously published [19]–[21]. A summary of basic demographic characteristics and study samples sizes is provided in Table 1.

**Table 1:** Demographic data summary.

	Guineans (Guinea-Bissau)		Gambians		African-Americans		European Americans/Argentinians	
	Cases(N = 321)	Controls(N = 346)	Cases(N = 258)	Controls(N = 271)	Cases(N = 295)	Controls(N = 179)	Cases(N = 237)	Controls(N = 144)
Age, mean ± SD, (years)	37.08±13.73	35.58±12.40	33.34±13.63	29.09±13.14	45.48±17.86	51.91±21.24	39.20±17.61	40.67±20.28
Sex (%)								
Male	60.44	49.71	69.38	69.74	65.97	83.53	52.20	62.12
Female	35.56	50.29	30.62	30.26	34.02	16.47	47.7	37.0
Ethnicity (%)								
Balanta	15.26	19.36	**-**	**-**	**-**	**-**	**-**	**-**
Fulani	14.95	11.56	12.18	6.27	**-**	**-**	**-**	**-**
Jola	-	-	21.43	34.12				
Mancanha	8.10	12.43	-	-	**-**	**-**	**-**	**-**
Mandinka	7.48	7.51	39.08	32.55	**-**	**-**	**-**	**-**
Manjaco	19.00	9.54	-	-	**-**	**-**	**-**	**-**
Papel	20.25	29.77	-	-				
Wolof	-	-	11.34	13.33	**-**	**-**	**-**	**-**
Other	14.95	9.83	15.97	13.73	**-**	**-**		

#### Guinea-Bissau

This case-control study was conducted at the Bandim Health Project (BHP), a demographic surveillance site in Bissau, the capital of Guinea-Bissau. The incidence of PTB in this area is among the highest in the world, 470/100,000. Our Guinean cohort consisted of Papel (25%), Balanta (17%), Manjaco (14%), Fulani (13%), Mancanha (10%), Mandinka (7%), and other ethnicities (12%). Cases were residents or long-term guests of Bissau, aged greater than 15 years and newly diagnosed with PTB using three sputum examinations for acid fast bacteria or clinical criteria by the World Health Organization's definition of active pulmonary TB [22]. No culture confirmation of TB was available in Bissau during the study period, as facilities were destroyed during a civil war; 218/321 (68%) cases were smear positive. Patients with newly diagnosed TB were enrolled when they started antitubercular treatment at local health centers. During the inclusion period from November 2003 to November 2005, 438 TB patients were screened at local health centers: 344 subjects met inclusion criteria and provided written informed consent, and from these we could obtain 321 DNA samples.

Healthy controls were recruited from the study area from May 2005 to November 2005. A random sample of 200 houses was selected from the database of all subjects living in the study area; houses with a recorded case of TB within the past 2 years were excluded from the sampling. Exclusion criteria for controls included the presence of cough for more than 2 weeks, history of TB and TB in the household within the last 2 years to avoid households with a high-risk of active TB. The composition of the case and control samples was different in terms of sex and ethnicity. These differences are due to the sampling strategy as controls were derived from healthy nuclear families; hence more healthy married couples were collected, whereas TB patients are more often males. The ethnic differences are due to willingness of healthy subjects to give blood, which was not the same across the ethnic groups, whereas most TB patients agreed to participate in the study regardless of their ethnic background. All controls were unrelated to cases. Analyses were also performed excluding cases who only had clinical diagnoses to test for the effect of these samples on our results (Table S1).

All subjects were interviewed by field assistants, using a standardized questionnaire on ethnicity, environmental factors and prior exposure to TB. Permission to perform HIV tests was obtained for cases but not for controls, as requiring HIV testing would have negatively impacted participation in the study. Venous blood samples were collected from all subjects. Ethical approval was granted by the ‘Unidade de Coordenacao de Estudos e Pesquisas em material de Saude’ (Ministry of Health) in Guinea-Bissau. All adults and children's guardians signed a written informed consent to the study.

#### The Gambia

Between June 2002 and October 2004, PTB cases and their household contacts were enrolled in a prospective cohort study in the Greater Banjul region of The Gambia, where about 750,000 people live, representing more than 50% of the total Gambian population (http://www.columbia.edu/~msj42/index.htm). Our Gambian cohort consisted of Mandinka (36%), Jola (28%), Wolof (12%), Fulani (9%), and other ethnicities (15%). According to WHO 2007 burden estimates, the incidence of TB in The Gambia is 258/100,000 and 11% of new TB cases are HIV positive (http://www.who.int/globalatlas/predefinedReports/TB/PDF_Files/gmb.pdf). Recruitment took place at the major government TB clinic and the Medical Research Council (MRC) outpatient clinic, and consisted of sputum smear positive pulmonary TB cases at least 15 years old, who had at least one household contact living with them [19]. HIV positive patients were excluded from the study, and all included patients had two positive sputum smear samples for acid-fast bacilli and Mtb isolated upon culture. An index case was defined as the first TB case identified in a household. Household contact controls were defined as individuals living the majority of the time on the same compound as the index TB case, sharing meals and identifying a common household head. Written informed consent was obtained from all subjects, including parents/guardians for minors. The study was approved by the combined Gambia Government/MRC National Ethics Committee of The Gambia.

Subjects were interviewed by field assistants, using a standardized questionnaire on ethnicity, environmental factors and prior exposure to TB. Permission to perform HIV tests was asked for cases but not for controls. Venous blood samples were collected from all subjects, and from these, 258 case DNA samples and 271 control DNA samples (unrelated to cases) were genotyped for analyses. All samples were archived in the National Gambian DNA Bank and used in compliance with the bank guidelines [23].

#### African-Americans, European ancestry Americans and Argentineans

Participants were ascertained through the North Carolina or South Carolina TB Control Programs, U.S.A., or as patients at the outpatient clinic at F.J. Muñiz Hospital in Buenos Aires, Argentina, between 2002 and 2006. Criteria for inclusion as TB cases were: a) age 14 years of age or older and culture-confirmed PTB, or b) younger than 14 years old and either culture-confirmed or clinically diagnosed PTB that included a positive tuberculin skin test plus an infiltrate or hilar adenopathy on chest x-ray. Some of the cases only had clinical diagnoses (7% for the African-American and European-American cases and 4% for the Argentinean cases). To test for the effect of these samples on the results they were excluded in a set of sensitivity analyses (Table S1).

Individuals were eligible to participate if TB had been diagnosed in the past, or if they were currently receiving TB treatment. All TB cases remained eligible if they also had a diagnosis of extrapulmonary TB. Family members of eligible TB cases, who themselves had a history of TB, were enrolled as part of a multi-case family if review of their records established diagnosis of either pulmonary or extrapulmonary TB. Thus, a small portion of our study subjects enrolled as part of a multi-case family had extrapulmonary TB only.

Severity of TB disease was assessed by presence of acid-fast bacilli (AFB) in sputum smears (7% African-Americans, 4% European-Americans/Argentineans) or x-ray evidence of cavitary lesions. We attempted to document HIV status through medical record review for all subjects. However, participation in this study did not require that the individual authorize review of HIV test results.

Unaffected individuals who were in close contact with cases, such as household contacts such as spouses and partners, and relatives such as parents and siblings, were enrolled as controls. Written informed consent was obtained from all subjects or their legal representatives before participation in the study. Human experimentation guidelines of the U.S. Department of Health and Human Services and those of the participating research institutions were followed. The protocol was IRB-approved at Duke University Medical Center, the North and South Carolina Departments of Public Health (USA), Centro de Educación Médica e Investigaciones Clínicas “Norberto Quirno” (CEMIC), the F.J. Muñiz Hospital, Buenos Aires, Argentina, and the University of Miami Miller School of Medicine.

### DNA extraction and genotyping

**Figure 1:**
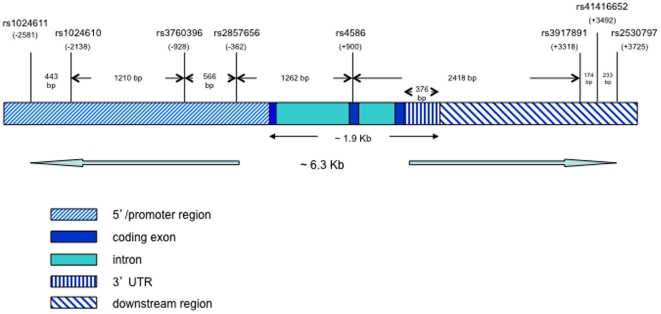
*MCP1* Gene Structure and SNP Positions. *MCP1* is oriented 5′ to 3′ with SNPs indicated above the gene with rs numbers and position relative to coding sequence start site.

SNPs were selected based on either being associated with PTB in previous studies (rs1024611, rs2857656 and rs4586) or being a haplotype tagging SNP in the *MCP1* gene [14], [24], [25]. Tags were selected from HapMap phase III samples: African-Americans (from the SW USA), Africans (Yoruba, Maasai, and Luhya), Mexicans (from Los Angeles, USA) and Northern and Western Europeans (Centre d'Etude du Polymorphisme Humain (CEPH) family samples from Utah, USA) (http://www.hapmap.org). To focus analysis on common variants for which these samples have the greatest statistical power to detect effects, SNPs with minor allele frequency greater or equal to 0.1 and located in a region extending 3 kb on either side of the gene were identified from the HapMap and Genome Variation Server databases and were grouped into bins of highly correlated SNPs (r^2^ greater than or equal to 0.80). A single “tagSNP” was selected from each bin for genotyping. A summary of the SNPs examined is provided (Figure 1).

All DNA samples were extracted using a standard salting-out procedure (Guinea-Bissau and The Gambia) or the Puregene method from Gentra systems (African-Americans and European-Americans/Argentineans). DNA purities were estimated spectrophotometrically, and final concentrations were determined by PicoGreen. One of the SNPs (rs1024611) genotyped in Guinea-Bissau and The Gambia samples was genotyped by TaqMan assay (ABI, Applera International Inc, Foster City, CA, USA) in 10 µl reaction volume, using the Rotor-Gene 3000 (Corbett Robotics Pty Ltd, Brisbane, Queensland, Australia) and the ABI 7500 real-time PCR system. Fluorescence curves were analyzed with the Rotor-Gene Software version 6 and the 7500 Sequence Detection Software version 1.2.1 for allelic discrimination. The remaining SNPs in all populations (Guinea-Bissau, The Gambia, African-Americans, and European-Americans/Argentineans) were genotyped using TaqMan assays on an ABI 7900 HT with genotype calling performed using ABI SDS software. All SNPs used in this study had genotyping call rates of 95% or better (mean call rates of 98%) and quality control duplicate sample match rates of 100%.

### Bioinformatics Tools

SNP base pair (bp) position and function was identified using the SNPper (http://snpper.chip.org) database NCBI Build 35.1 (Figure 1). The HapMap database (http://www.hapmap.org) was used to obtain linkage disequilibrium (LD) and genotype information from the Yoruba and CEPH populations.

### Statistical Methods

All analyses were performed separately for the four cohorts (Guinea-Bissau, The Gambia, African-Americans, and European-Americans/Argentineans). For Guinea-Bissau and The Gambia tests for deviations from Hardy-Weinberg Equilibrium (HWE) were performed using PLINK statistical software [26]. Tests for deviations from HWE in African-Americans and European-Americans/Argentineans were calculated using genetic data analysis (GDA) software using one case and one control from each pedigree [27]. Statistical significance for these analyses was determined using Fisher's exact test.

Pairwise LD was characterized, standard summary statistics D′ and r^2^, and haplotype frequencies were calculated using HaploView statistical software [28]. Haplotype blocks were assigned, using the D′ confidence interval algorithm created by Gabriel et al. 2002 [29]. Haplotype analyses were performed Guinea Bissau and The Gambia with 3 and 8-marker sliding windows using PLINK software. Analyses were run using haplotype-bases association test with generalized linear models (GLMs) adjusting for age, sex, and ethnic group. Haplotype analyses only included common haplotypes (haplotype frequency ≥0.05) and statistical significance was assessed with 1,000 permutations to generate empirical p values.

Single locus tests of association in Guinea-Bissau and The Gambia were performed using logistic regression models with PLINK software [26] assuming an additive genetic model. Odds ratios (ORs) and confidence intervals (CI) were reported for all statistical models. Confounding by age, ethnicity, and sex was evaluated in logistic regression models; for inclusion in the final model we required that a change in effect size for the SNP be greater than or equal to 0.05. As a result, all regression models were performed with an adjustment for age, ethnicity, and sex. Unadjusted models are presented in Table S2. A 2-degree of freedom genotypic test of association was also performed with PLINK software for Guinea-Bissau and The Gambia for model-free tests of association.

For African-Americans and European-Americans/Argentineans single locus additive genotypic tests of association were performed with generalized estimating equations (GEE) using the independence correlation matrix implemented in STATA 11.0 statistical software (College Station, TX). GEE performs a valid test of gene×gene and gene×environment interactions in mixed family and case-control data [30]. We also performed GEE analyses adjusting for the potential confounders, age and sex for all analyses. For European-Americans/Argentineans we did the analyses two ways. First, we performed the analyses in these two cohorts separately; second, we incorporated ascertainment site in the models, using the combined data (Table S3). Because there was no evidence of a recruitment site effect between Argentinian and European-American cohorts, we pooled these samples in subsequent analyses.

Two locus interaction analyses were performed to test for interactions between *MCP1* and *IL12B* based on the observation in previous studies that a functional relationship exists between *MCP1* promoter polymorphisms and IL12B concentrations [14]. The main effects for the polymorphisms examined in *IL12B* in our cohort have been previously published in Morris et al. 2011 [18] (a list of those variants is provided in Table S4). Two locus interactions were examined between *MCP1* and *IL12B* polymorphisms with a MAF greater than 0.05 within a population and were performed with logistic regression for Guinea Bissau and The Gambia and with GEE for African-Americans and European-Americans/Argentineans using STATA 11.0 statistical software (College Station, TX). These analyses were performed adjusting for the same covariates used in single locus tests of associations. A Bonferroni correction for multiple testing was used to adjust for multiple testing for single locus and gene-gene interactions. Examination of allele and genotype frequency differences for cases with and without HIV indicated no evidence for significant differences between the two groups in any of the populations examined; as a result analyses were performed pooling cases with and without HIV. Sensitivity analyses were also performed by excluding clinically diagnosed TB cases for Guinea Bissau, African-Americans, and European-Americans/Argentineans. Overall, these analyses showed no difference in the association results.

Finally, in order to assess the concordance of results across study populations meta-analyses of single SNP associations were performed using PLINK software [26]. Meta-analyses were run both including and excluding the European/Argentinean study population.

## Results

### Guinea-Bissau and The Gambia

**Figure 2:**
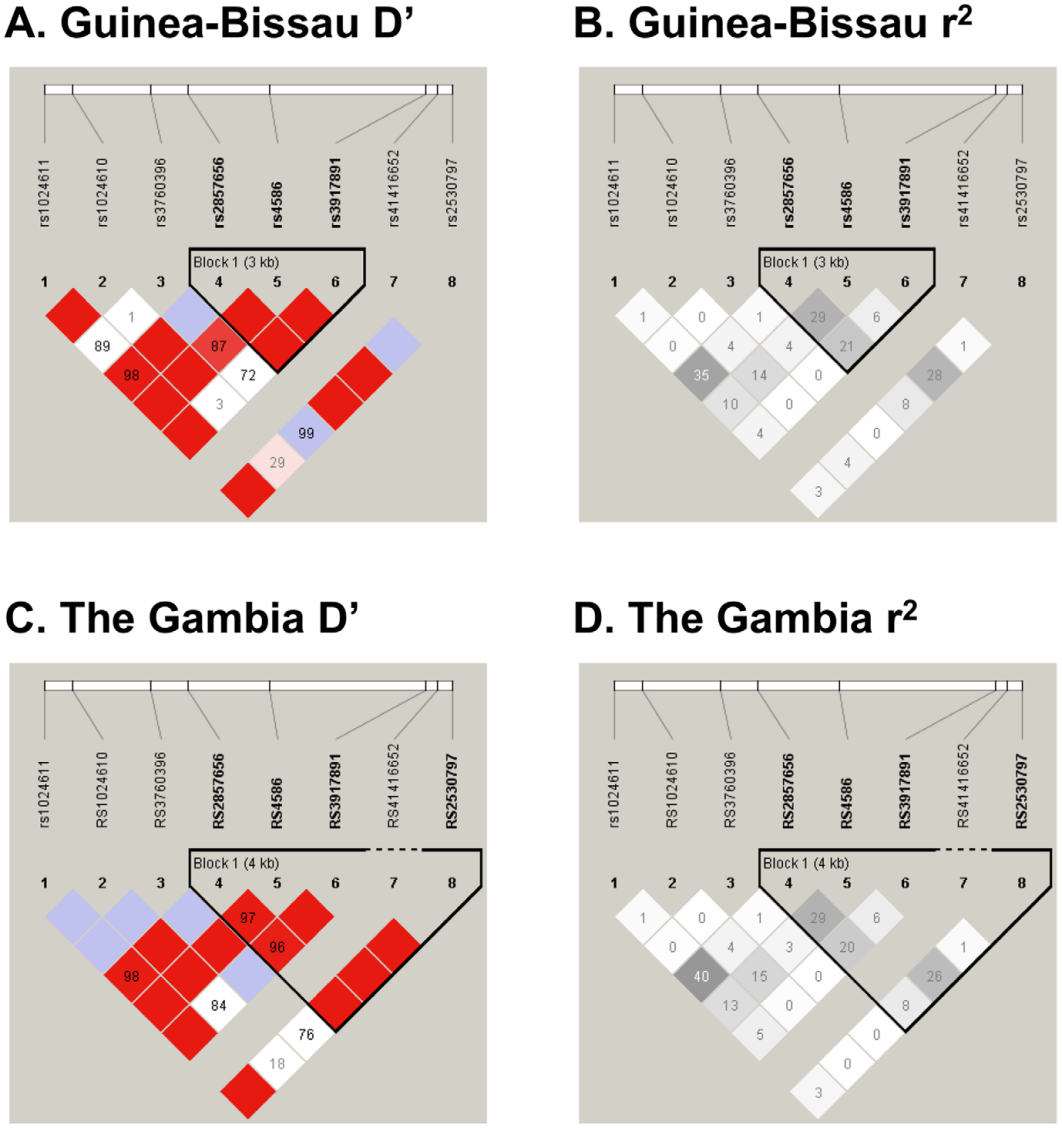
Guinea-Bissau and The Gambia HaploView plots for controls (*MCP1*). LD plots are presented for Guinea Bissau controls (A and B) and for The Gambia controls (C and D) including both D′ and r^2^. All figures are oriented 5′ to 3′, right to left, relative to the gene orientation on the minus strand. D′ (shades of red) and r^2^ (shades of black) are indicated in percentages within squares in the LD plots, with solid blocks without numbers indicating D′ and r^2^ = 1. Strong LD is indicated by red or dark gray, while pink and light gray and white indicate uninformative and low confidence values, respectively. LD Blocks were created with the default algorithm in HaploView that creates 95% confidence bounds on D′ considered being in strong LD where 95% of the comparisons made are informative. The haplotype blocks were created using HaploView program, version 4.1.

Single locus tests of association did not identify a statistically significant association at any of the SNPs examined with either the logistic regression model for allelic association (Table 2) or using the 2 degree of freedom genotypic test (results not shown). A borderline association was observed in the Guinea-Bissau population but not in The Gambia at *MCP1* marker rs2530797 with an OR = 1.39, 95% CI [0.95–2.02], p = 0.088 (Table 2). The association became less statistically significant for the dominant model (CC (referent) versus CT & TT, OR = 1.25, 95% CI [0.33–4.78], p = 0.742) and the recessive model (results not shown). Detailed analyses of the previously associated SNPs rs1024611 and rs2857656 demonstrated a borderline significant association in the Guinea-Bissau population for rs1024611 under the dominant model (AA (referent) versus AG & GG, OR = 1.36, 95% CI [0.96–1.93], p = 0.079) (Table 3). SNP rs2530797 was in LD with both previously associated SNPs rs1024611 (D′ = 1; r^2^ = 0.03) and rs2857656 (D′ = 1; r^2^ = 0.08) in the Guinea-Bissau population (Figure 2A and 2B). No statistically significant haplotype associations were found in the Guinean and Gambian cohorts (Table S5).

**Table 2:** Guineans and Gambians single locus association results adjusted for age, ethnicity and sex.

Population	Marker	Genotype	Genotype Counts		OR^2^	95% CI		Additivep-Value
			Cases	Controls		Lower	Upper	
Guineans	rs1024611*	GG	17	21	1.23	0.92	1.63	0.163
		AG	123	103				
		AA	174	217				
	rs1024610^1^	TT	2	0	0.64	0.36	1.15	0.137
		AT	22	38				
		AA	288	305				
	rs3760396	CC	0	0	1.00	0.46	2.24	0.999
		CG	16	16				
		GG	293	323				
	rs2857656	CC	56	63	0.97	0.76	1.23	0.786
		CG	150	158				
		GG	107	119				
	rs4586	TT	35	29	1.05	0.81	1.36	0.702
		CT	129	143				
		CC	149	169				
	rs3917891^1^	TT	1	10	0.89	0.60	1.35	0.604
		CT	69	70				
		CC	244	261				
	rs41416652	CC	0	0	-	-	-	-
		CT	1	0				
		TT	309	342				
	rs2530797	CC	5	7	1.39	0.95	2.02	**0.088**
		CT	71	59				
		TT	240	277				
Gambians	rs1024611*	GG	18	15	1.00	0.75	1.34	0.991
		AG	80	93				
		AA	138	144				
	rs1024610^1^	TT	1	0	0.93	0.51	1.70	0.818
		AT	24	29				
		AA	219	230				
	rs3760396^1^	CC	0	0	1.59	0.61	4.18	0.346
		CG	11	8				
		GG	231	253				
	rs2857656	CC	44	50	0.94	0.72	1.22	0.623
		CG	116	129				
		GG	80	81				
	rs4586	TT	22	20	1.04	0.78	1.38	0.798
		CT	96	109				
		CC	126	131				
	rs3917891	TT	6	6	0.86	0.59	1.25	0.427
		CT	56	63				
		CC	181	187				
	rs41416652	CC	0	0	-	-	-	-
		CT	0	0				
		TT	247	266				
	rs2530797^1^	CC	5	3	1.02	0.63	1.63	0.951
		CT	38	44				
		TT	204	216				

*Indicates a statistically significant deviation from HWE in either cases or controls.

1 A dominant model was used to calculate the association p value because the number of individuals in the rare homozygous class was below 5 in cases, controls, or both.

2 OR is for additive model except for those instances where a dominant model was used.

**Table 3:** Additive, dominant, and recessive regression models for SNPs previously associated in published studies adjusted for age, ethnicity and sex.

Population	Marker	Model	OR	95% CI		p-Value
				Low	Upper	
Guineans	rs1024611	AA(referent) vs AG vs GG	1.23	0.92	1.63	0.163
		AA(referent) vs AG&GG	1.36	0.96	1.93	**0.079**
		AA&AG(referent) vs GG	0.96	0.46	2.01	0.915
	rs2857656	GG(referent) vs GC vs CC	0.97	0.76	1.23	0.786
		GG(referent) vs GC&CC	0.97	0.68	1.38	0.851
		GG&GC(referent) vs CC	0.94	0.61	1.46	0.792
Gambians	rs1024611	AA(referent) vs AG vs GG	1.00	0.75	1.34	0.991
		AA(referent) vs AG&GG	0.94	0.65	1.35	0.744
		AA&AG(referent) vs GG	1.28	0.62	2.65	0.499
	rs2857656	GG(referent) vs GC vs CC	0.94	0.72	1.22	0.623
		GG(referent) vs GC&CC	0.90	0.62	1.33	0.611
		GG&GC(referent) vs CC	0.94	0.58	1.50	0.783
African-Americans	rs1024611	AA(referent) vs AG vs GG	1.27	0.83	1.93	0.272
		AA(referent) vs AG&GG	1.31	0.81	2.10	0.270
		AA&AG(referent) vs GG	1.33	0.33	5.41	0.687
	rs2857656	GG(referent) vs GC vs CC	1.12	0.60	2.09	0.717
		GG(referent) vs GC&CC	1.21	0.76	1.92	0.418
		GG&GC(referent) vs CC	0.66	0.35	1.25	0.204
European-Americans/Argentineans	rs1024611	AA(referent) vs AG vs GG	1.18	0.63	2.23	0.599
		AA(referent) vs AG&GG	1.78	0.59	5.35	0.303
		AA&AG(referent) vs GG	0.96	0.36	2.53	0.933
	rs2857656	GG(referent) vs GC vs CC	1.12	0.60	2.09	0.717
		GG(referent) vs GC&CC	1.82	0.61	5.47	0.285
		GG&GC(referent) vs CC	0.84	0.33	2.17	0.723

Interaction analyses revealed evidence for a gene-gene interaction between *MCP1* SNP rs2857656 and *IL12B* SNP rs2288831 (Table S6, OR_INT_ = 1.90, 95% CI [1.31–2.77], p = 0.001) in Guinea-Bissau; however, this result was not in the same direction in The Gambia, despite being statistically significant (p = 0.024). Neither of these results was significant after correction for multiple testing.

### African-Americans and European-Americans/Argentineans

**Figure 3:**
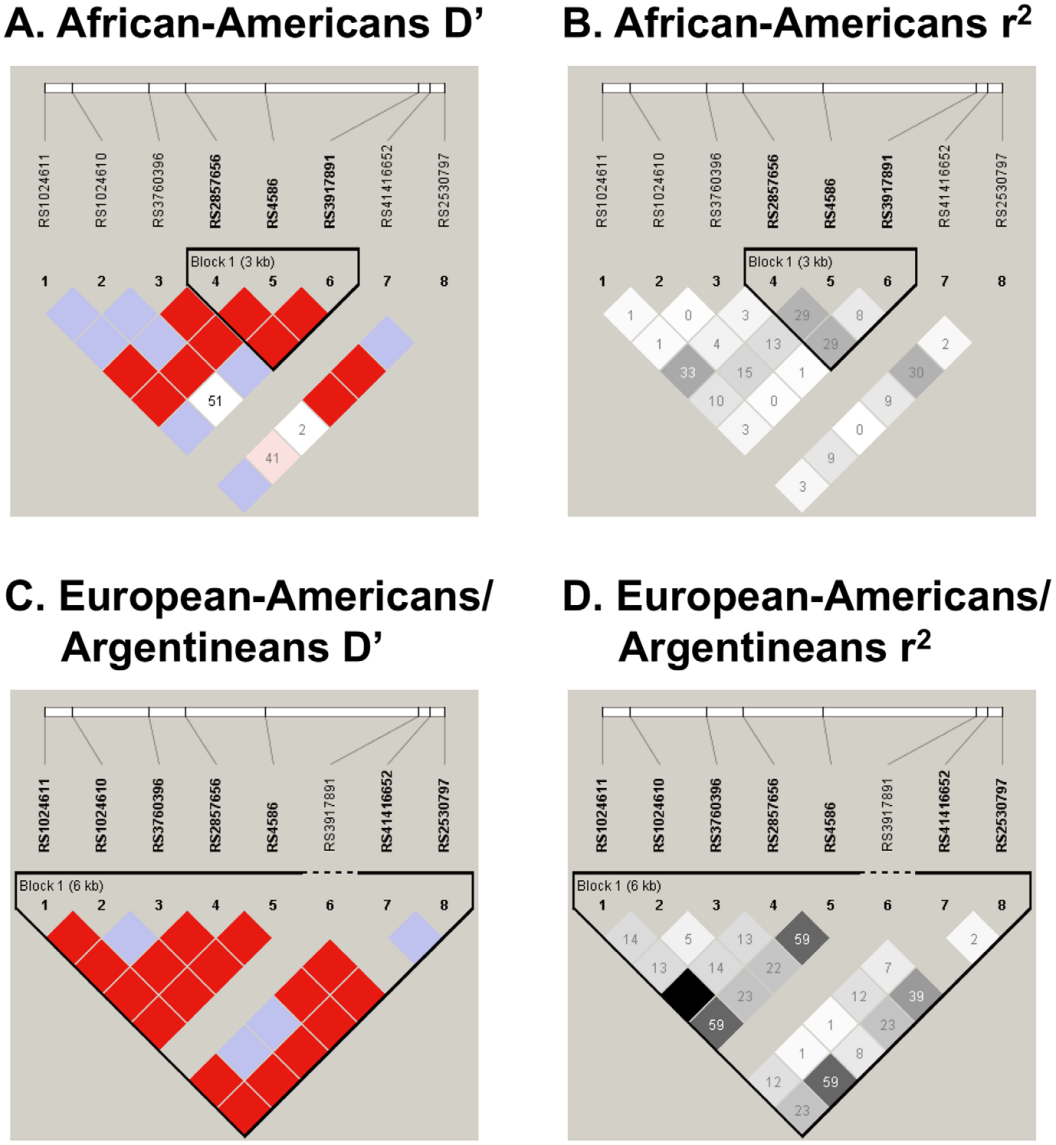
African-Americans and European-Americans/Argentineans HaploView plots for controls (*MCP1*). LD plots are presented for African-American controls (A and B) and for European-American/Argentinean controls (C and D) including both D′ and r^2^. All figures are oriented 5′ to 3′, right to left, relative to the gene orientation on the minus strand. D′ (shades of red) and r^2^ (shades of black) are indicated in percentages within squares in the LD plots, with solid blocks without numbers indicating D′ and r^2^ = 1. Strong LD is indicated by red or dark gray, while pink and light gray and white indicate uninformative and low confidence values, respectively. LD Blocks were created with the default algorithm in HaploView that creates 95% confidence bounds on D′ considered being in strong LD where 95% of the comparisons made are informative. The haplotype blocks were created using HaploView program, version 4.1.

Single locus tests of association did not identify a statistically significant association at any of the SNPs in the African-American and European-American/Argentinean populations (Table 4). LD plots for African-American and for European-American/Argentinean controls are shown in Figure 3, respectively 3A–B and 3C–D. There were no statistically significant interactions between *MCP1* and *IL12B* in European-Americans/Argentineans; however, in African-Americans there was one statistically significant interaction between *MCP1* rs3917891 and *IL12B* rs11574790 (OR_INT_ = 0.28, 95% CI [0.13–0.65], p = 0.003) (Table S6). None of the associations were statistically significant after correction for multiple testing.

**Table 4:** African-Americans and European-Americans/Argentineans single locus association results using an additive GEE model adjusted for age and sex.

Population	Marker	Genotype	Genotype Counts		OR^2^	95% CI		Additivep-Value
			Cases	Controls		Low	Upper	
African-Americans	rs1024611^1^	GG	8	4	1.31	0.83	2.05	0.243
		AG	91	49				
		AA	189	123				
	rs1024610^1^	TT	2	0	1.07	0.54	2.13	0.848
		AT	36	22				
		AA	250	151				
	rs3760396^1^	CC	1	0	1.29	0.69	2.39	0.425
		CG	26	14				
		GG	259	158				
	rs2857656	CC	38	27	0.99	0.71	1.38	0.947
		CG	140	81				
		GG	111	65				
	rs4586	TT	42	24	0.80	0.58	1.11	0.179
		CT	124	60				
		CC	121	88				
	rs3917891^1^	TT	7	4	0.72	0.43	1.20	0.207
		CT	59	50				
		CC	222	117				
	rs41416652	CC	1	0	-	-	-	-
		CT	4	1				
		TT	284	174				
	rs2530797^1^	CC	11	1	1.56	0.91	2.69	0.100
		CT	73	39				
		TT	202	133				
European-Americans/Argentinians	rs1024611	GG	68	32	1.18	0.63	2.23	0.599
		AG	95	53				
		AA	70	55				
	rs1024610	TT	5	6	0.70	0.19	2.55	0.585
		AT	56	35				
		AA	171	102				
	rs3760396^1^	CC	3	5	0.55	0.23	1.29	0.168
		CG	44	32				
		GG	183	103				
	rs2857656	CC	69	33	1.12	0.60	2.09	0.717
		CG	96	54				
		GG	71	54				
	rs4586	TT	44	39	1.37	0.68	2.79	0.379
		CT	102	57				
		CC	83	45				
	rs3917891	TT	0	0	-	-	-	-
		CT	2	2				
		CC	228	140				
	rs41416652^1^	CC	12	2	1.54	0.61	3.88	0.356
		CT	47	17				
		TT	176	121				
	rs2530797	CC	14	17	1.02	0.45	2.31	0.971
		CT	97	53				
		TT	117	69				

Statistical models for African-Americans included an adjustment for age and sex and European-Americans/Argentineans also included an adjustment for ascertainment site.

1 A dominant model was used to calculate the association p value because the number of individuals in the rare homozygous class was below 5 in cases, controls, or both.

2 OR is for additive model except for those instances where a dominant model was used.

The strongest evidence for concordant single SNP association across study populations was for *MCP1* rs2530797, (OR_meta_ = 1.30, random effect meta-analysis p = 0.051). Cochrane's Q statistic (p≥0.36) and the I^2^ index of heterogeneity (I≤3.36, scale 0–100) indicated little evidence for heterogeneity for this association across study populations for these SNPs. The results did not change when removing the European American/Argentinian sample (data not shown).

## Discussion

In the present study we examined eight SNPs in *MCP1* for association with PTB in two African populations, one African-American population, and populations of European ancestry from North and South America. We focused on variants that had been shown to be associated with either increased or decreased TB risk, although previous studies were inconsistent, possibly reflecting differences both in genetic structure and phenotype definitions.

We did not observe any statistically significant association at the SNPs studied in Guineans, Gambians, African-Americans and European-Americans/Argentineans. Examination of all previously associated SNPs did not provide evidence for association in any of our populations. We observed a statistically significant interaction between *MCP1* and *IL12B* in the West African cohorts; however, the association was in the opposite direction in the two populations, indicating that this is likely to be spurious.

Our data is in contrast to the majority of that published to date (Table S7). Specifically for the SNPs we genotyped that have been previously examined we found:

1. rs1024611 (−2581A/G) associations with PTB have been reported in Mexican, Korean, Ghanaian, Zambian, Tunisian, Moroccan and Peruvian cohorts [14], [24], [31]–[34]. This SNP, originally reported by Flores-Villanueva et al., promoted subsequent genetic studies of *MCP1* and TB [14]. However, while in Ghanaians the “G” allele and the “AG+GG” genotypes were found to confer protection from PTB (OR = 0.81) [24], in a Moroccan sample only the “GG” genotype showed the same effect (OR = 0.35) [33]; in all other populations typed the “G” allele associated with increased risk (e.g. OR = 2.63 in Koreans, OR = 1.29 in Peruvians) [14], [34]. We also observed a trend in the direction of increased risk. The heterogeneous effect of the “G” allele was also reported in a meta-analysis by Feng et al. (Table S7) [35].
2. rs2857656 (−362G/C): in Ghanaians, the “C” allele and “CG+CC” genotypes were associated with protection from disease (OR = 0.83) [24]. In the same population, when SNPs rs1024611 and rs2857656 were combined in an extended haplotype including a 14 bp insertion/deletion, rs3917887 (haplotype: “rs1024611 G- rs2857656 C- rs3917887 del”), the protective effect size increased (OR = 0.78) [36], though the combination of genetic data with functional assays indicated that the role of rs1024611 was small at best. According to Thye et al. rs2857656 was the variant driving the protective effect originally observed in Ghanaians (see Table S7). Our data provide no evidence for association of rs2857656.
3. rs4586 (+900C/T): the “C” allele and the “CC+TC” genotypes were associated with increased risk of TB (OR = 1.34 and 1.94, respectively), in a pediatric cohort from Northern China that was heterogeneous with respect to phenotype definitions, ranging from pulmonary TB (35%) to extra-pulmonary TB (26%) and TB meningitis (39%) [25]; moreover, the association was only found in males. This SNP was not significantly associated with TB in the Ghanaian study [24] nor in ours.

Analyses of extended haplotypes in our Guineans and Gambians, encompassing polymorphisms associated with protection from TB in Ghanaians [36], did not reveal any association signal.

Taken together our data do not support any of these previously associated variants.

We also tested for interactions between *MCP1* and *IL12B* based on prior experimental evidence supporting an interaction between these two genes [14] and our own recent study showing an *IL12B* association with PTB susceptibility in the same African ancestry samples examined in this paper [18]. Although we did observe some weak evidence for risk-modulating interactions in Guineans, Gambians and African-Americans, none of the *MCP1-IL12B* effects remained statistically significant after correction for multiple testing, and, most importantly, they were not always in the same direction. In conclusion, our data indicate that there was no evidence for a genetic interaction between *MCP1* and *IL12B* with respect to susceptibility to PTB.

In our study there were aspects that are worth discussing. We used two different ascertainment centers for the collection of European-ancestry samples, one from the Southeastern U.S. and another from Argentina, and they included a small number of HIV-positive cases as well as cases of extrapulmonary TB. In order to account for this we included ascertainment site as a variable in our models and performed sensitivity analyses excluding HIV-positive and extrapulmonary TB cases. Site, HIV status and extrapulmonary TB did not influence the significance of our results. Both the Guinea-Bissau and The Gambia population samples showed evidence for confounding by ethnic groups, but we dealt with this by adjustment for ethnicity. Finally, there were some limitations regarding power to detect effect sizes previously found. Within our Gambian cohort we had approximately 80% power to detect OR ranging between <0.49 or >1.8 with a MAF of 0.20, while, in our Guinea Bissau cohort with the same MAF we had 80% power to detect OR ranging between <0.53 >1.70. Although we were underpowered to detect associations of the effect size reported in the Ghanaian population (OR = 0.81 for rs1024611 and OR = 0.83 for rs2857656) [24], we failed to detect effects in the same direction as those previously published for both rs1024611 and rs2857656 in our West African samples. These discordant results are cause for caution in interpreting the role of *MCP1* in TB susceptibility. However, it is possible that the lack of replication is due to unmeasured variables interacting with the *MCP1* SNPs. Although we would have been able to have increased power by pooling the Guinea Bissau and Gambian cohorts, several studies have shown significant genetic heterogeneity across African populations, even within limited geographical areas [20], [37], and for this reason we chose not to pool our populations but instead to meta-analyze the results. Meta-analysis did not detect statistically significant, robust results across the studies. Our findings of interactions in opposite directions in these two cohorts support the decision not to pool.

In conclusion, this study did not replicate associations with TB previously observed in *MCP1*. Although this is a highly relevant candidate gene, our data indicate that the effect of genetic variation within *MCP1* is not clear cut and additional studies will be needed to elucidate its role in TB susceptibility.

## Supporting Information

Table S1 **Sensitivity analysis including only culture confirmed or smear positive TB cases adjusted for covariates.**
(DOC)

Table S2 **Single locus tests of association in Guineans and Gambians unadjusted for age, sex and ethnicity.**
(DOC)

Table S3 **African-Americans and European-Americans/Argentineans single locus association results using an additive GEE model unadjusted for age and sex.**
(DOC)

Table S4 **IL12B polymorphisms examined in MCP1×IL12B interaction analyses.**
(DOC)

Table S5 **Guineans and Gambians 8 and 3 marker sliding window haplotype analysis.**
(DOC)

Table S6 **Top (p<0.05) gene×gene interaction (MCP1×IL12B) results across cohorts.** Two locus interactions are presented between MCP1 and IL12B polymorphisms with a MAF greater than 0.05 within a population. These analyses were performed with logistic regression for Guinea Bissau and The Gambia and with GEE for African-Americans and European-Americans/Argentineans using STATA 11.0 statistical software (College Station, TX) and were performed adjusting for the same covariates used in single locus tests of associations. A Bonferroni correction for multiple testing was used to adjust for multiple testing for single locus and gene×gene interactions. The gene×gene interactions results are presented according to increasing p values.
(DOC)

Table S7 **Genetic association of MCP1 variants with pulmonary tuberculosis.**
(DOC)
