# Supplementary material for: MCP1 SNPs and Pulmonary Tuberculosis in Cohorts from West Africa, the USA and Argentina: Lack of Association or Epistasis with IL12B Polymorphisms
Source: PLoS One. 2012 Feb 27;7(2):e32275. doi: 10.1371/journal.pone.0032275 (PMC3288089; doi:10.1371/journal.pone.0032275)
Supplement: Table S1 — Sensitivity analysis including only culture confirmed or smear positive TB cases adjusted for covariates. (DOC) [file pone.0032275.s001.doc]

**Table S1. Sensitivity analysis including only culture confirmed or smear positive TB cases adjusted for covaria**tes

| **Population** | **Marker** | **OR** | **95% CI** | | **Additive**  **p-Value** |
| --- | --- | --- | --- | --- | --- |
|  |  |  | **Low** | **Upper** |  |
| Guineans^1^ | rs1024611 | 1.20 | 0.89 | 1.61 | 0.236 |
|  | rs1024610 | 0.72 | 0.39 | 1.31 | 0.272 |
|  | rs3760396 | 1.26 | 0.57 | 2.79 | 0.575 |
|  | rs2857656 | 0.90 | 0.70 | 1.16 | 0.425 |
|  | rs4586 | 1.28 | 0.98 | 1.65 | 0.068 |
|  | rs3917891 | 0.68 | 0.44 | 1.06 | 0.082 |
|  | rs41416652 | - | - | - | - |
|  | rs2530797 | 1.35 | 0.93 | 1.97 | 0.113 |
| African-Americans^2^ | rs1024611 | 1.23 | 0.76 | 1.98 | 0.407 |
|  | rs1024610 | 1.08 | 0.51 | 2.28 | 0.837 |
|  | rs3760396 | 1.45 | 0.75 | 2.83 | 0.269 |
|  | rs2857656 | 0.98 | 0.70 | 1.38 | 0.924 |
|  | rs4586 | 0.82 | 0.59 | 1.14 | 0.238 |
|  | rs3917891 | 0.65 | 0.37 | 1.16 | 0.146 |
|  | rs41416652 | - | - | - | - |
|  | rs2530797 | 1.69 | 0.94 | 3.03 | 0.077 |
| European-Americans/Argentineans^2^ | rs1024611 | 1.18 | 0.63 | 2.23 | 0.600 |
|  | rs1024610 | 0.69 | 0.19 | 2.53 | 0.575 |
|  | rs3760396 | 0.68 | 0.28 | 1.66 | 0.395 |
|  | rs2857656 | 1.12 | 0.60 | 2.09 | 0.721 |
|  | rs4586 | 1.37 | 0.68 | 2.78 | 0.383 |
|  | rs3917891 | 2.61 | 0.13 | 50.97 | 0.528 |
|  | rs41416652 | 1.27 | 0.49 | 3.34 | 0.622 |
|  | rs2530797 | 1.02 | 0.45 | 2.32 | 0.967 |

^1^ Only sputum examination

^2^ Only culture confirmation
