# Supplementary material for: MCP1 SNPs and Pulmonary Tuberculosis in Cohorts from West Africa, the USA and Argentina: Lack of Association or Epistasis with IL12B Polymorphisms
Source: PLoS One. 2012 Feb 27;7(2):e32275. doi: 10.1371/journal.pone.0032275 (PMC3288089; doi:10.1371/journal.pone.0032275)
Supplement: Table S2 — Single locus tests of association in Guineans and Gambians unadjusted for age, sex and ethnicity. (DOC) [file pone.0032275.s002.doc]

**Table S2. Single locus tests of association in Guineans and Gambians unadjusted for age, sex and ethnicity**

| **Population** | **Marker** | **MA^1^** | **OR** | **95% CI** | | **Additive**  **p-Value** |
| --- | --- | --- | --- | --- | --- | --- |
|  |  |  |  | **Lower** | **Upper** |  |
| Guineans | rs1024611* | G | 1.23 | 0.95 | 1.58 | 0.114 |
|  | rs1024610 | T | 0.74 | 0.44 | 1.23 | 0.244 |
|  | rs3760396 | C | 1.10 | 0.54 | 2.23 | 0.803 |
|  | rs2857656 | C | 1.00 | 0.80 | 1.24 | 0.995 |
|  | rs4586 | T | 1.11 | 0.88 | 1.40 | 0.377 |
|  | rs3917891 | T | 0.83 | 0.60 | 1.16 | 0.283 |
|  | rs41416652 | T | - | - | - | - |
|  | rs2530797 | C | 1.23 | 0.89 | 1.71 | 0.214 |
| Gambians | rs1024611* | G | 1.02 | 0.77 | 1.35 | 0.894 |
|  | rs1024610 | T | 0.95 | 0.55 | 1.64 | 0.849 |
|  | rs3760396 | C | 1.51 | 0.60 | 3.81 | 0.387 |
|  | rs2857656 | C | 0.94 | 0.73 | 1.21 | 0.624 |
|  | rs4586 | T | 1.00 | 0.76 | 1.32 | 0.990 |
|  | rs3917891 | T | 0.95 | 0.67 | 1.35 | 0.771 |
|  | rs41416652 | T | - | - | - | - |
|  | rs2530797 | C | 1.02 | 0.68 | 1.53 | 0.912 |
