# Supplementary material for: MCP1 SNPs and Pulmonary Tuberculosis in Cohorts from West Africa, the USA and Argentina: Lack of Association or Epistasis with IL12B Polymorphisms
Source: PLoS One. 2012 Feb 27;7(2):e32275. doi: 10.1371/journal.pone.0032275 (PMC3288089; doi:10.1371/journal.pone.0032275)
Supplement: Table S3 — African-Americans and European-Americans/Argentineans single locus association results using an additive GEE model unadjusted for age and sex. (DOC) [file pone.0032275.s003.doc]

**Table S3. African-Americans and European-Americans/Argentineans single locus association results using an additive GEE model unadjusted for age and sex**

| **Population** | **Marker** | **MA** | **OR** | **95% CI** | | **Additive**  **p-Value** |
| --- | --- | --- | --- | --- | --- | --- |
|  |  |  |  | **Low** | **Upper** |  |
| African-Americans | rs1024611 | G | 1.19 | 0.83 | 1.70 | 0.347 |
|  | rs1024610 | T | 0.91 | 0.53 | 1.56 | 0.731 |
|  | rs3760396 | C | 1.21 | 0.63 | 2.34 | 0.564 |
|  | rs2857656 | C | 0.93 | 0.71 | 1.23 | 0.615 |
|  | rs4586 | T | 0.82 | 0.63 | 1.08 | 0.157 |
|  | rs3917891 | T | 1.39 | 0.96 | 2.02 | 0.078 |
|  | rs41416652 | T | - | - | - | - |
|  | rs2530797 | C | 1.47 | 0.99 | 2.16 | 0.054 |
| Argentineans | rs1024611 | G | 1.15 | 0.60 | 2.19 | 0.675 |
|  | rs1024610 | T | 0.66 | 0.18 | 2.48 | 0.541 |
|  | rs3760396 | C | 0.51 | 0.19 | 1.39 | 0.189 |
|  | rs2857656 | C | 1.09 | 0.58 | 2.05 | 0.796 |
|  | rs4586 | T | 1.32 | 0.64 | 2.72 | 0.453 |
|  | rs3917891 | T | - | - | - | - |
|  | rs41416652 | C | 1.52 | 0.69 | 3.36 | 0.305 |
|  | rs2530797 | C | 1.09 | 0.47 | 2.53 | 0.844 |
| European-Americans | rs1024611 | G | 0.99 | 0.58 | 1.69 | 0.969 |
|  | rs1024610 | T | 0.88 | 0.41 | 1.88 | 0.741 |
|  | rs3760396 | C | 0.87 | 0.39 | 1.94 | 0.736 |
|  | rs2857656 | C | 0.95 | 0.56 | 1.63 | 0.859 |
|  | rs4586 | T | 0.90 | 0.52 | 1.56 | 0.715 |
|  | rs3917891 | T | - | - | - | - |
|  | rs41416652 | C | 1.40 | 0.45 | 4.31 | 0.557 |
|  | rs2530797 | C | 1.16 | 0.62 | 2.17 | 0.646 |
| European-Americans/Argentineans | rs1024611 | G | 1.72 | 1.10 | 2.68 | 0.016 |
|  | rs1024610 | T | 1.60 | 0.74 | 3.49 | 0.232 |
|  | rs3760396 | C | 0.56 | 0.27 | 1.18 | 0.126 |
|  | rs2857656 | C | 1.68 | 1.08 | 2.60 | 0.020 |
|  | rs4586 | T | 1.91 | 1.17 | 3.11 | 0.010 |
|  | rs3917891 | T | - | - | - | - |
|  | rs41416652 | C | 2.05 | 1.07 | 3.91 | 0.030 |
|  | rs2530797 | C | 0.61 | 0.36 | 1.04 | 0.072 |

*Statistical models for European-Americans/Argentineans included an adjustment for ascertainment site.
