# Supplementary material for: MCP1 SNPs and Pulmonary Tuberculosis in Cohorts from West Africa, the USA and Argentina: Lack of Association or Epistasis with IL12B Polymorphisms
Source: PLoS One. 2012 Feb 27;7(2):e32275. doi: 10.1371/journal.pone.0032275 (PMC3288089; doi:10.1371/journal.pone.0032275)
Supplement: Table S4 — IL12B polymorphisms examined in MCP1×IL12B interaction analyses. (DOC) [file pone.0032275.s004.doc]

**Table S4. *IL12B* polymorphisms examined in *MCP1* x *IL12B* interaction analyses**

| ***Gene*** | **Marker** | **Position** | **Role** |
| --- | --- | --- | --- |
|  |  |  |  |
|  |  |  |  |
| *IL12B*  Chromosome 5q31.1-q33.1 | rs17860508 | 158692783 | Promoter |
|  | rs6894567 | 158689546 | Intron 1 |
|  | rs3212220 | 158686773 | Intron 1 |
|  | rs10631390 | 158683690 | Intron 2 |
|  | rs2288831 | 158682591 | Intron 3 |
|  | rs919766 | 158680142 | Intron 4 |
|  | rs2421047 | 158678885 | Intron 5 |
|  | rs11574790 | 158676424 | Intron 6 |
|  | rs3212227 | 158675528 | 3' UTR |
