# Supplementary material for: MCP1 SNPs and Pulmonary Tuberculosis in Cohorts from West Africa, the USA and Argentina: Lack of Association or Epistasis with IL12B Polymorphisms
Source: PLoS One. 2012 Feb 27;7(2):e32275. doi: 10.1371/journal.pone.0032275 (PMC3288089; doi:10.1371/journal.pone.0032275)
Supplement: Table S5 — Guineans and Gambians 8 and 3 marker sliding window haplotype analysis. (DOC) [file pone.0032275.s005.doc]

**Table S5. Guineans and Gambians 8 and 3 marker sliding window haplotype analysis**

**A. Guineans**

| **Markers in Haplotype** | | | | | | | | **Global P** |
| --- | --- | --- | --- | --- | --- | --- | --- | --- |
| rs1024611 | rs1024610 | rs3760396 | rs2857656 | rs4586 | rs3917891 | rs41416652 | rs2530797 | 0.260 |
| rs1024611 | rs1024610 | rs3760396 |  |  |  |  |  | 0.232 |
| rs1024610 | rs3760396 | rs2857656 |  |  |  |  |  | 0.778 |
| rs3760396 | rs2857656 | rs4586 |  |  |  |  |  | 0.506 |
| rs2857656 | rs4586 | rs3917891 |  |  |  |  |  | 0.342 |
| rs4586 | rs3917891 | rs41416652 |  |  |  |  |  | 0.544 |
| rs3917891 | rs41416652 | rs2530797 |  |  |  |  |  | 0.332 |

**B. Gambians**

| **Markers in Haplotype** | | | | | | | | **Global P** |
| --- | --- | --- | --- | --- | --- | --- | --- | --- |
| rs1024611 | rs1024610 | rs3760396 | rs2857656 | rs4586 | rs3917891 | rs41416652 | rs2530797 | 0.929 |
| rs1024611 | rs1024610 | rs3760396 |  |  |  |  |  | 0.792 |
| rs1024610 | rs3760396 | rs2857656 |  |  |  |  |  | 0.995 |
| rs3760396 | rs2857656 | rs4586 |  |  |  |  |  | 0.968 |
| rs2857656 | rs4586 | rs3917891 |  |  |  |  |  | 0.821 |
| rs4586 | rs3917891 | rs41416652 |  |  |  |  |  | 0.750 |
| rs3917891 | rs41416652 | rs2530797 |  |  |  |  |  | 0.711 |
