# Supplementary material for: MCP1 SNPs and Pulmonary Tuberculosis in Cohorts from West Africa, the USA and Argentina: Lack of Association or Epistasis with IL12B Polymorphisms
Source: PLoS One. 2012 Feb 27;7(2):e32275. doi: 10.1371/journal.pone.0032275 (PMC3288089; doi:10.1371/journal.pone.0032275)
Supplement: Table S6 — Top (p<0.05) gene×gene interaction (MCP1×IL12B) results across cohorts. Two locus interactions are presented between MCP1 and IL12B polymorphisms with a MAF greater than 0.05 within a population. These analyses were performed with logistic regression for Guinea Bissau and The Gambia and with GEE for African-Americans and European-Americans/Argentineans using STATA 11.0 statistical software (College Station, TX) and were performed adjusting for the same covariates used in single locus tests of associations. A Bonferroni correction for multiple testing was used to adjust for multiple testing for single locus and gene×gene interactions. The gene×gene interactions results are presented according to increasing p values. (DOC) [file pone.0032275.s006.doc]

**Table S6. Top (p < 0.05) gene x gene interaction (*MCP1*x*IL12B*) results across cohorts**

| **Population** | **Interaction** | **OR** | **95% CI** | | **p-Value** |
| --- | --- | --- | --- | --- | --- |
|  |  |  | **Lower** | **Upper** |  |
| Guineans | rs2288831(*IL12B*)*rs2857656(*MCP1*) | 1.90 | 1.31 | 2.77 | 0.001 |
|  | rs2421047(*IL12B*)*rs2857656(*MCP1*) | 1.80 | 1.23 | 2.62 | 0.002 |
|  | rs2530797(*MCP1*)*rs919766(*IL12B*) | 3.17 | 1.55 | 6.49 | 0.002 |
|  | rs2857656(*MCP1*)*rs3212227(*IL12B*) | 1.89 | 1.26 | 2.83 | 0.002 |
|  | rs2857656(*MCP1*)*rs3212220(*IL12B*) | 1.74 | 1.21 | 2.51 | 0.003 |
|  | rs10631390(*IL12B*)*rs2857656(*MCP1*) | 1.62 | 1.12 | 2.34 | 0.010 |
|  | rs2288831(*IL12B*)*rs3917891(*MCP1*) | 1.95 | 1.09 | 3.48 | 0.024 |
|  | rs3212227(*IL12B*)*rs3917891(*MCP1*) | 2.06 | 1.10 | 3.85 | 0.024 |
|  | rs1024610(*MCP1*)*rs3917891(*MCP1*) | 0.09 | 0.01 | 0.78 | 0.029 |
|  | rs3212220(*IL12B*)*rs3917891(*MCP1*) | 1.82 | 1.03 | 3.21 | 0.040 |
|  | rs1024611(*MCP1*)*rs2421047(*IL12B*) | 1.54 | 1.00 | 2.35 | 0.048 |
| Gambians | rs2288831(*IL12B*)*rs2857656(*MCP1*) | 0.64 | 0.44 | 0.94 | 0.024 |
|  | rs1024611(*MCP1*)*rs2288831(*IL12B*) | 0.64 | 0.42 | 0.96 | 0.033 |
|  | rs1024611(*MCP1*)*rs3212220(*IL12B*) | 0.66 | 0.44 | 0.98 | 0.04 |
|  | rs2857656(*MCP1*)*rs3212220(*IL12B*) | 0.68 | 0.47 | 0.99 | 0.047 |
| African-Americans | rs11574790(*IL12B*)*rs3917891(*MCP1*) | 0.28 | 0.13 | 0.65 | 0.003 |
|  | rs3917891(*MCP1*)*rs919766(*IL12B*) | 0.42 | 0.19 | 0.90 | 0.027 |
|  | rs11574790(*IL12B*)*rs2857656(*MCP1*) | 1.97 | 1.06 | 3.66 | 0.031 |

**The gene x gene interactions are listed according to increasing P values**
