# Supplementary material for: MCP1 SNPs and Pulmonary Tuberculosis in Cohorts from West Africa, the USA and Argentina: Lack of Association or Epistasis with IL12B Polymorphisms
Source: PLoS One. 2012 Feb 27;7(2):e32275. doi: 10.1371/journal.pone.0032275 (PMC3288089; doi:10.1371/journal.pone.0032275)
Supplement: Table S7 — Genetic association of MCP1 variants with pulmonary tuberculosis. (DOC) [file pone.0032275.s007.doc]

**Table S7. Genetic association of *MCP1* variants with pulmonary tuberculosis**

| **SNP/variant** | **Population** | **Samples** | **Allele/Genotype/**  **Haplotype** | **Increased risk**  OR (95% CI)  p-value | **Decreased risk**  OR (95% CI)  p-value | **Reference** |
| --- | --- | --- | --- | --- | --- | --- |
| rs9889296 **^a^**  (-11822G/A, 9241 bp from rs1024611) | Ghana | 2010 TB cases  2346 healthy controls (2217 tuberculin-positive, 129 tuberculin-negative) | A  AG+AA  (additive model) |  | 0.84 (0.77-0.92)  p = 0.001  0.85 (0.77-0.92) **^b^**  p = 0.0012 | [1] |
| rs1024611  (-2581A/G) | Mexico  Korea | Mexico:  435 TB cases  334 healthy tuberculin-positive controls  176 healthy tuberculin-negative controls  Korea:  129 TB cases  162 healthy controls | G (TB vs PPD+)  G (TB vs PPD-)  GG (TB vs PPD+)  GG (TB vs PPD-)  G  GG | 2.43 (1.96-3.02)  p = 0.0003  2.45 (1.88-3.19)  p = 0.0026  5.4 (3.4-8.6) **^c^**  p = 0.00001  5.5 (3.2-9.5) **^c^**  p = 0.00001  2.63 (1.85-3.73)  p = 0.00001  6.9 (3.4-14.1) **^c^**  p = 3.2x10^-8^ |  | [2] |
| rs1024611 | Zambia | 46 TB cases  115 healthy tuberculine-positive controls | AG (vs AA) | 2.8 (1.4-5.8)  p = 0.01 **^d^** |  | [3] |
| rs1024611 | Mexico  Peru | Mexico:  193 TB cases **^e^**  243 healthy tuberculin-positive controls  Peru:  701 TB cases **^e^**  796 healthy tuberculin-positive controls | G  GG  **^f^**  G  GG **^f^** | 1.75 (1.3-2.3)  p = 0.0082  2.66 (1.47-4.79)  p = 0.001  1.29 (1.1-1.5)  p = 0.007  1.43 (1.02-2.0)  p = 0.036 |  | [4] |
| rs1024611 | Tunisia | 168 PTB cases **^g^**  55 EPTB cases  150 healthy controls | G (PTB vs controls) | 1.83 (1.26-2.66)  p = 0.0007 **^d^** |  | [5] |
| rs1024611 | Pakistan | 110 asymptomatic household contacts, BCG vaccinated, 75% TST+ (HC)  86 endemic community controls, BCG vaccinated, 46% TST+ (EC) | GG (HC vs EC) | χ^2^ = 8.98 **^h^**  p = 0.003 |  | [6] |
| rs1024611 **^a^** | Ghana |  | G  AG+GG  (additive model) |  | 0.81 (0.73-0.91)  p = 0.0012  0.81 (0.73-0.91) **^b^**  p = 0.0018 | [1] |
| rs1024611 | Morocco | 337 TB patients  204 healthy controls | GG |  | 0.35 (0.13-0.86)  p = 0.04 | [7] |
| rs1024611  Meta-analysis | China (Chongqing, Hong Kong, Shandong), Ghana, India, Korea, Mexico, Peru, South Africa (Admixed)  Asians  Latin-Americans  Africans | 4676 TB cases  5260 controls | G  GG  GG+AG  G  G | 1.51 (1.11-2.04)  p = 0.008  1.66 (1.19-2.33)  p = 0.003  1.53 (1.07-2.17)  p = 0.018  1.64 (1.07-2.51)  p = 0.024  2.00 (1.23-3.25)  p = 0.005 | 0.82 (0.74-0.90)  p = 0.000 | [8] |
| rs1024611 | Russia | 1440 TB cases  1529 healthy controls |  | No association  p = 0.86 |  | [1] |
| rs1024611 | Hong Kong | 412 TB cases  465 healthy controls |  | No association  p = 0.61 |  | [9] |
| rs1024611 | Iran | 142 PTB cases  166 healthy subjects |  | No association  p = 0.493 |  | [10] |
| rs1024611 | India | 81 PTB HIV+  31 EPTB HIV+  155 PTB HIV-  206 healthy controls |  | No association  (p values not indicated) |  | [11] |
| rs1024611 | Brazil | 18 miliary/meningeal TB  28 PTB control subjects  29 controls with latent *M.tuberculosis* infection PPD+ |  | No association  (p values from 0.74 to 0.92) |  | [12] |
| rs1024611  rs1024610 (-2138A/T) | Brazil | 280 TB cases  347 healthy controls |  | No association  (p values not indicated) |  | [13] |
| **rs2857656 ^a^**  **(-362G/C, 2219 bp from rs1024611)** | Ghana |  | C  CG+CC  (additive model) |  | 0.83 (0.76-0.90)  p = 0.00017  0.83 (0.76-0.91) **^b^**  p = 0.00023 | [1] |
| rs1024611/ rs2857656 | Ghana |  | Haplotype G/C |  | 0.82 (0.74-0.92)  p = 0.0003 | [1] |
| rs3917887  (int1del554-567, 3211 bp from rs1024611, 992 bp from rs2857656 | Ghana | 2010 TB cases  2346 healthy controls (2217 tuberculin-positive, 129 tuberculin-negative) | del  ins/del + del/del  (additive model) |  | 0.85 (0.78-0.92)  p = 0.00098  0.84 (0.77-0.92) **^b^**  p = 0.00098 | [14] |
| rs1024611/ rs2857656/ rs3917887 | Ghana |  | Haplotype G/C/del |  | 0.78 (0.69-0.87)  p = 0.00002 | [14] |
| rs4586 **^i^**  (+900C/T, 3481 bp from rs1024611) | China (Han from Northern China) | 301 TB cases **^l^**  338 tuberculine-negative controls | C (males)  CC+TC (males) | 1.34 (1.01-1-79)  p = 0.045  1.94 (1.06-3.56)  p = 0.029 |  | [15] |
| rs4586 | Ghana |  |  | No association after correction  (p values not indicated) |  | [1] |
| rs991804 **^a^**  (+5356C/T, 7937 bp from rs1024611) | Ghana |  | T  CT+TT  (additive model) |  | 0.84 (0.77-0.92)  p = 0.00093  0.85 (0.78-0.92) **^b^**  p = 0.0011 | [1] |
| rs2857654  rs1024611  rs1024610  rs3760399  rs4586  rs2530797 | South Africa (Admixed) | 431 TB cases  482 healthy controls | Single polymorphism and haplotype analysis | No association  (p values from 0.11 to 1) |  | [16] |

PTB = pulmonary TB

EPTB = extrapulmonary TB

BCG = Bacille Calmette-Guérin

TST = tuberculin skin test

PPD = purified protein derivative

We decided not to reference articles written in Chinese only, that we could not critically assess

**^a^** Although the authors found several SNPs associated with resistance from TB and some of them were in LD, the protection effect resulted was driven by rs28576565 (-362 G/C)

**^b^** OR trend, estimates of an additive genetic model

**^c^** The authors observed a dosage effect of the *MCP1* susceptibility allele G in both populations

**^d^** Uncorrected p-value

**^e^** BCG-vaccinated patients

**^f^** It is not observed a dose effect of allele G as in non-BCG-vaccinated patients (Flores-Villanueva et al., 2005); the authors concluded that BCG vaccination is a modifier of the allele effect. The risk of developing TB is increased by the joint effect of rs1024611 GG genotype and the -1607 (rs1799750) 2G/2G genotype of the matrix metalloproteinase 1 (*MMP-1*) gene: in Mexicans OR = 3.59 (1.54-8.33), p = 0.003 and in Peruvians OR = 3.9 (2.56-5.95), p = 0.0001

**^g^** Genotype frequencies of the -2581 G/A, rs1024611 SNP not in Hardy-Weinberg equilibrium in the PTB group

**^h^** OR not indicated; the authors suggested an effect of GG genotype in increasing the risk of latent TB infection

**^i^** rs4586 allele C and rs1024611 allele G are in LD (D’=0.975, r^2^=0.9) (China Han Beijing CHB dataset in HapMap PHASE 3)

**^l^** TB cases: 105 PTB (34.9%), 78 EPTB (25.9%), 118 TB meningitis (39.2%)

Reference List

1. Thye T, Nejentsev S, Intemann CD, Browne EN, Chinbuah MA, et al (2009) MCP-1 promoter variant -362C associated with protection from pulmonary tuberculosis in Ghana, West Africa. Hum Mol Genet 18**:** 381-388.

2. Flores-Villanueva PO, Ruiz-Morales JA, Song CH, Flores LM, Jo EK, et al (2005) A functional promoter polymorphism in monocyte chemoattractant protein-1 is associated with increased susceptibility to pulmonary tuberculosis. J Exp Med 202**:** 1649-1658.

3. Buijtels PC, van de Sande WW, Parkinson S, Petit PL, van der Sande MA, et al (2008) Polymorphism in CC-chemokine ligand 2 associated with tuberculosis in Zambia. Int J Tuberc Lung Dis 12**:** 1485-1488.

4. Ganachari M, Ruiz-Morales JA, Gomez de la Torre Pretell JC, Dinh J, Granados J, et al (2010) Joint effect of MCP-1 genotype GG and MMP-1 genotype 2G/2G increases the likelihood of developing pulmonary tuberculosis in BCG-vaccinated individuals. PLoS One 5**:** e8881.

5. Ben-Selma W, Harizi H, Boukadida J (2011) MCP-1 -2518 A/G functional polymorphism is associated with increased susceptibility to active pulmonary tuberculosis in Tunisian patients. Mol Biol Rep 38**:** 5413-5419.

6. Hussain R, Ansari A, Talat N, Hasan Z, Dawood G (2011) CCL2/MCP-I genotype-phenotype relationship in latent tuberculosis infection. PLoS ONE 6**:** e25803.

7. Arji N, Busson M, Iraqi G, Bourkadi JE, Benjouad A, et al (2012) The MCP-1 (CCL2) -2518 GG genotype is associated with protection against pulmonary tuberculosis in Moroccan patients. J Infect Dev Ctries 6**:** 73-78.

8. Feng WX, Flores-Villanueva PO, Mokrousov I, Wu XR, Xiao J, et al (2011) CCL2-2518 (A/G) polymorphisms and tuberculosis susceptibility: a meta-analysis. Int J Tuberc Lung Dis Dec 2.

9. Chu SF, Tam CM, Wong HS, Kam KM, Lau YL, et al (2007) Association between RANTES functional polymorphisms and tuberculosis in Hong Kong Chinese. Genes Immun 8**:** 475-479.

10. Naderi M, Hashemi M, Karami H, Moazeni-Roodi A, Sharifi-Mood B, et al (2011) Lack of Association between rs1024611 (-2581 A/G) Polymorphism in CC-chemokine Ligand 2 and Susceptibility to Pulmonary Tuberculosis in Zahedan, Southeast Iran. Prague Med Rep 112**:** 272-278.

11. Alagarasu K, Selvaraj P, Swaminathan S, Raghavan S, Narendran G, et al (2009) CCR2, MCP-1, SDF-1a & DC-SIGN gene polymorphisms in HIV-1 infected patients with & without tuberculosis. Indian J Med Res 130**:** 444-450.

12. Sterling TR, Martire T, de Almeida AS, Ding L, Greenberg DE, et al (2007) Immune function in young children with previous pulmonary or miliary/meningeal tuberculosis and impact of BCG vaccination. Pediatrics 120**:** e912-e921.

13. Jamieson SE, Miller EN, Black GF, Peacock CS, Cordell HJ, et al (2004) Evidence for a cluster of genes on chromosome 17q11-q21 controlling susceptibility to tuberculosis and leprosy in Brazilians. Genes Immun 5**:** 46-57.

14. Intemann CD, Thye T, Forster B, Owusu-Dabo E, Gyapong J, et al (2011) MCP1 haplotypes associated with protection from pulmonary tuberculosis. BMC Genet 12**:** 34.

15. Feng WX, Mokrousov I, Wang BB, Nelson H, Jiao WW, et al (2011) Tag SNP Polymorphism of CCL2 and Its Role in Clinical Tuberculosis in Han Chinese Pediatric Population. PLoS ONE 6**:** e14652.

16. Moller M, Nebel A, Valentonyte R, van Helden PD, Schreiber S, et al (2009) Investigation of chromosome 17 candidate genes in susceptibility to TB in a South African population. Tuberculosis (Edinb) 89**:** 189-194.
